# Association of Socioeconomic Position with Incident Hypertension Hospitalization and Blood Pressure Control among Participants in the Coronary Artery Risk Development in Young Adults Study

**DOI:** 10.1093/ajh/hpaf231

**Published:** 2025-11-26

**Authors:** Lama Ghazi, Cora E Lewis, Byron C Jaeger, Catarina I Kiefe, Kathryn Foti, Kelley P Gabriel, Norrina B Allen, Marwah Abdalla, Phillip D Levy, Shakia T Hardy, Paul Muntner

**Affiliations:** Department of Epidemiology, School of Public Health, University of Alabama at Birmingham, Birmingham, AL, United States; Department of Epidemiology, School of Public Health, University of Alabama at Birmingham, Birmingham, AL, United States; Department of Biostatistics and Data Science, Wake Forest University School of Medicine, Winston-Salem, NC, United States; Department of Population, Quantitative Health Sciences University of Massachusetts Chan Medical School, Worcester, MA, United States; Department of Epidemiology, School of Public Health, University of Alabama at Birmingham, Birmingham, AL, United States; Department of Epidemiology, School of Public Health, University of Alabama at Birmingham, Birmingham, AL, United States; Department of Preventive Medicine, Northwestern University Feinberg School of Medicine, Chicago, IL, United States; Department of Medicine, Columbia University Irving Medical Center, New York, NY, United States; Department of Emergency Medicine and Integrative Biosciences Center, Wayne State University, Detroit, MI, United States; Department of Epidemiology, Gillings School of Global Public Health, University of North Carolina at Chapel Hill, Chapel Hill, NC, United States; Department of Epidemiology, School of Public Health, University of Alabama at Birmingham, Birmingham, AL, United States; Perisphere Real-World Evidence, Austin, TX, United States

**Keywords:** hypertension, hospitalization, socioeconomic position, blood pressure control

## Abstract

**Background:**

The rate of hypertension hospitalizations is increasing among US adults. Individuals with low socioeconomic position are more likely to have high blood pressure (BP), which may increase their risk of hypertension hospitalization and adverse post-discharge outcomes.

**Methods:**

We analyzed data from the Coronary Artery Risk Development in Young Adults (CARDIA) cohort study, which enrolled 5115 adults aged 18 to 30 years from 4 urban US communities in 1985-1986. Hospitalizations were identified by self-report during study exams and annual interviews, with hypertension hospitalizations determined through medical record review, through August 2020. Socioeconomic position included education, family income, having private health insurance, and neighborhood deprivation assessed at the last study visit prior to the hypertension hospitalization. Uncontrolled BP (≥140/90 mmHg) was determined at the first CARDIA study visit after hypertension hospitalization.

**Results:**

Overall, 67 CARDIA participants were hospitalized for hypertension. The hazard ratio of hypertension hospitalization among participants who had less than high school vs high school or more education was 3.12 [95% CI, 1.78-5.48], whose family income was <$25,000 vs ≥$25,000 was 2.43 [95% CI, 1.44-4.11], who had no private vs private insurance was 2.58 [95% CI, 1.56-4.28] and those in tertile 3 vs tertile 1 of neighborhood deprivation index (most vs least deprived) was 3.06 [95% CI, 1.23-7.58]. Among 46 participants who attended a CARDIA study visit following hospital discharge, 23 (50%) had uncontrolled BP.

**Conclusion:**

Adults with low socioeconomic position were more likely to be hospitalized for hypertension. Uncontrolled BP was common following hypertension hospitalization.

## Graphical abstract

**Graphical Abstract:**
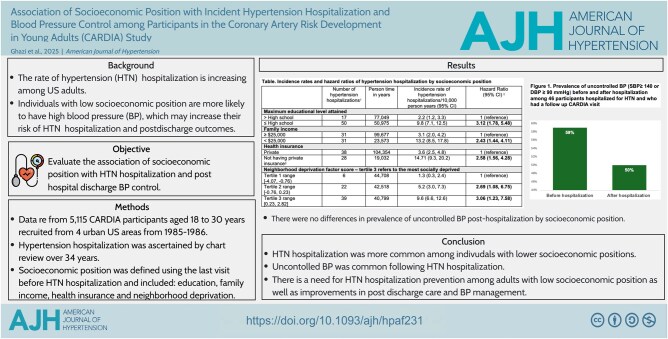

## Introduction

The rate of hypertension hospitalization, with and without end-organ damage, has increased in the United States over the past 20 years.^1–4^ For example, among Medicare fee-for-service beneficiaries aged 65 years or older, the annual age-adjusted hospitalization rate for hypertension, regardless of end-organ damage, increased from 51.5 to 125.9 per 100,000 beneficiary-years between 1999 and 2019.^1^ These adjusted trends indicate that the rising hospitalization rates were not explained by population aging within this group.

Hypertension and uncontrolled blood pressure (BP) are more common among adults with low socioeconomic position, characterized by lower educational attainment, lower household income, living in a disadvantaged neighborhood, and lack of health insurance or reliance on public insurance.^5–9^ Furthermore, among individuals who present to the emergency department with high BP, those residing in low-income areas are more likely than their counterparts residing in high-income areas to be admitted for inpatient management.^10^ Hypertension hospitalization is associated with increased cardiovascular risk following discharge.^1–4^,^11^,^12^ Having controlled BP after discharge has been associated with lower risk of cardiovascular disease events.^13–15^ Determining the association of socioeconomic position with hypertension hospitalization and BP control after discharge may facilitate identification of high-risk populations and the development of tailored interventions to prevent hypertension hospitalizations and improve BP control in the post-discharge period.

We analyzed data from the Coronary Artery Risk Development in Young Adults (CARDIA) study to evaluate the association of socioeconomic position with incident hypertension hospitalization and post-hospital discharge BP control, from young adulthood to middle age.

## Methods

### Study population

Information on the CARDIA study design and participant selection has been previously published.^16^ Briefly, it is a prospective multi-center cohort study that enrolled adults aged 18-30 years in 1985-1986 from four urban areas (Birmingham, Alabama; Chicago, Illinois; Minneapolis, Minnesota, and Oakland, California) with the goal of understanding determinants, mechanisms, and outcomes of cardiovascular health and disease from young adulthood. The CARDIA study conducted follow-up in-person examinations at years 2, 5, 7, 10, 15, 20, 25, and 30 years after baseline with response rates of 91%, 86%, 81%, 79%, 74%, 72%, 72%, and 71% of the surviving cohort.^17^ Written informed consent was obtained from all study participants at each visit and the study’s protocol was approved by the institutional review board of each participating institution.

### Data collection timeline

Data on educational attainment level, annual family income, and health insurance were ascertained at the last CARDIA study visit attended by participants up until the year 30 exam (2015-2016). Data on neighborhood deprivation score, based on census data, were included through the year 20 exam (2005-2006) only, as the method of ascertainment changed to the American Community Survey in later exams and was not directly comparable (Supplementary Materials). Participants were contacted annually by telephone, mail, or electronic communication and asked whether they had been hospitalized or experienced major medical events, and medical records were retrieved for adjudication.^16^,^17^ Deaths were identified through attempted annual contact with the participant or a participant-designated proxy, as well as internet searches, and periodic linkage to the National Death Index. At the date of the analysis, adjudication of cardiovascular, kidney, and mortality outcomes was complete through August 31, 2020, and the current analysis included all data available up to that date.

### Hypertension hospitalization

For the present analysis, we reviewed medical records in which hypertension was reported as the primary reason or led to the primary reason (ie, target end-organ damage) for hospitalization (*n* = 150). One author (LG) reviewed admission notes, discharge summaries, and all hospitalization documents provided. A hospitalization was confirmed to be primarily for hypertension if the admitting diagnosis, hospital course, and discharge summary indicated that hypertension prompted the admission. LG did not have information on the participants’ socioeconomic positions at the time of reviewing the medical records. After review, 67 hospitalizations were confirmed to be primarily for hypertension, while the remaining 83 hospitalizations represented admissions for other comorbidities in which hypertension was a secondary diagnosis or occurred during hospitalization. Data on the date of hospitalization, vital signs on admission, target end-organ damage during hospitalization, BP on discharge, and whether antihypertensive medication was prescribed at discharge were abstracted from medical records. If the participant had multiple hypertension hospitalizations, we included only their first hospitalization.

### Target end-organ damage definition

Target end-organ damage was defined as a diagnosis of any of the following conditions as present on admission or having occurred during the hypertension hospitalization**:** acute kidney injury, aortic dissection, atrial fibrillation, angina, deep vein thrombosis, end-stage renal disease, heart failure, hypertensive encephalopathy, mesenteric ischemia, myocardial infarction, seizure, or stroke.^18^ These were identified using participants’ medical records.

### CARDIA BP assessment

Systolic BP (SBP) and diastolic BP (DBP) were measured by trained staff at each study visit following a standardized protocol (Supplementary Materials).^16^,^19^,^20^ Participants were asked to sit quietly for 5 min, with their feet flat on the floor and an appropriately sized cuff was used to measure BP. Three BP readings were taken at least 30 s apart and the average of the second and third readings was used to define BP at each study visit.

### Uncontrolled BP definition

Among participants hospitalized for hypertension, we assessed uncontrolled BP at the first CARDIA study visit attended after hypertension hospitalization. Uncontrolled BP was defined as having a mean SBP ≥140 or DBP ≥90 mmHg. These cut-points were selected based on the Seventh Report of the Joint National Committee on Prevention, Detection, Evaluation, and Treatment of High Blood Pressure (JNC7) guidelines, as the study visits analyzed occurred between 1985 and 2020, with the majority of follow-up taking place prior to the publication of the 2017 and 2025 American College of Cardiology/American Heart Association (ACC/AHA) BP guidelines.^21^ In a secondary analysis, uncontrolled BP was defined as having a mean SBP ≥130 or DBP ≥80 mmHg, in accordance with the 2025 ACC/AHA BP Guideline.^22^

### Socioeconomic position

We included four measures of socioeconomic position including ­educational attainment level (*≤*high school or >high school), annual family income (<$25,000 or ≥$25,000), health insurance (not having private insurance [Medicare or Medicaid, no insurance or Veterans Affairs [VA]] vs private insurance), and neighborhood deprivation score. Data on education, income, and health insurance were self-reported. Neighborhood deprivation was defined as a composite *z*-score of four factors that included median household income, proportion of population at or below the 150% federal poverty level, ­proportion of the population aged 25 years or greater with less than a high school education, and proportion of population aged 25 years or greater with a college degree or higher (Supplementary Materials).^23–26^ Due to the small number of participants who were hospitalized for hypertension, we categorized the neighborhood deprivation scores into tertiles instead of quartiles to ensure sufficient participants in each group for meaningful comparisons. The highest tertile represented the greatest neighborhood deprivation.

Data on medical history, laboratory values, cardiovascular events, kidney failure, and death were collected following standardized protocols and quality control procedures across study centers and examinations.

### Statistical analysis

Characteristics of participants who were and were not hospitalized for hypertension were estimated using mean and SD for continuous variables and frequencies and proportions for categorical variables. Continuous variables were assessed for normality prior to summarization. We calculated the incidence rate of hypertension hospitalization for each level of the four socioeconomic position variables. Participants who were hospitalized for hypertension were censored at time of hospitalization and those who were not were administratively censored on their date of death or the date of the last contact with the participant on or before the year 30 visit. Cox models were used to estimate the hazard ratio of hypertension hospitalization and socioeconomic position. Given the limited number of hypertension hospitalizations (*n* = 67), we prespecified a minimal adjustment set including age, sex, race, and study center to reduce overfitting and ensure model stability.

To assess BP and antihypertensive medication use following discharge for the hypertension hospitalization, we restricted the cohort to those who were hospitalized for hypertension and had a subsequent CARDIA visit, ie, only hospitalizations that occurred before the year 30 exam (2015-2016). We estimated mean SBP, mean DBP, and percentage taking antihypertensive medication assessed at the next CARDIA visit after hospitalization, overall and by socioeconomic position. We used a McNemar test to compare the prevalence of uncontrolled BP after vs before hypertension hospitalization. Poisson regression with robust variance estimates was used to study the association between socioeconomic position and uncontrolled BP at the next CARDIA visit after hypertension hospitalization. We fit the following models: Model 1 adjusted for age at time of hypertension hospitalization, sex, race, and CARDIA center; Model 2 adjusted for the variables in Model 1 and hypertension, diabetes, chronic kidney disease, smoking, alcohol consumption, body mass index, and low-density lipoprotein (LDL)-cholesterol at the CARDIA visit immediately before hospitalization.

In an exploratory analysis, we conducted an analysis of the composite outcome of adjudicated cardiovascular events and kidney failure, ascertained by CARDIA, occurring after the first hypertension-related hospitalization. This analysis was considered exploratory due to the modest number of participants hospitalized (Supplementary Materials). We estimated the incidence rate of the composite outcome by socioeconomic position. Cox proportional hazards models were used to estimate the association between socioeconomic position and the composite outcome, adjusting for age, sex, race, and study center. The proportional hazards assumption for all Cox regression models was evaluated using Schoenfeld residuals and log-minus-log survival plots and was not violated.

## Results

Over a maximum of 34 years of follow-up (median [25th, 75th percentile]: 29.8 [24.6, 30.2] years), 67 out of the 5115 (1.3%) CARDIA study participants had a hypertension hospitalization. Compared to participants who did not have a hypertension hospitalization, those who did were more likely to identify as having Black race (87% vs 51%), having been enrolled at the Birmingham site (49% vs 23%), have a history of hypertension at baseline (16% vs 4%), having a high school or less education (75% vs 43%), have a family income of less than $25,000 per year (46% vs 19%), have a family income of less than $25,000 per year (48% vs 29%), and were more likely to not have private insurance (42% vs 16%) (Table 1). A majority of participants who experienced a hypertension hospitalization (58%) lived in the most socially deprived neighborhoods, tertile 3 of neighborhood deprivation.

**Table 1.** Characteristics of CARDIA participants who did not have a hypertension hospitalization and those who had a hypertension hospitalization.

	Did not have a hypertension hospitalization	Had a hypertension hospitalization^a^
	*N* = 5047	*N* = 67
**Demographics at baseline (1985-1986)**		
**Age, years mean (SD)**	24.8 (3.7)	26.1 (3.7)
**Male sex, *n* (%)**	2293 (45%)	34 (51%)
**Black race, *n* (%)**	2579 (51%)	58 (87%)
**Study center at baseline, *n* (%)**		
**Birmingham**	1145 (23%)	33 (49%)
**Chicago**	1093 (22%)	15 (22%)
**Minnesota**	1395 (28%)	7 (10%)
**Oakland**	1414 (28%)	12 (18%)
**Health behaviors at baseline, *n* (%)**		
**Smoking status**		
**Never smoker**	2825 (56%)	31 (46%)
**Former smoker**	671 (13%)	5 (8%)
**Current smoker**	1515 (30%)	31 (46%)
**Drank alcohol in the past year**	4347 (86%)	57 (85%)
**Medical history at baseline, *n* (%)**		
**Hypertension**	184 (4%)	11 (16%)
**Diabetes**	31 (0.6%)	3 (5%)
**Chronic kidney disease**	208 (4%)	7 (10%)
**Anthropometrics at baseline, mean (SD)**		
**SBP, mmHg**	110.3 (10.9)	118.4 (12.3)
**DBP, mmHg**	68.5 (9.6)	74.5 (10.9)
**HR, beats/30 s**	34.7 (5.5)	34.4 (5.8)
**BMI, kg/m^2^**	24.5 (5.0)	26.6 (5.7)
**Laboratory values at baseline, median [25th percentile, 75th percentile]**		
**Triglycerides, mg/dL**	62 [45, 84]	64 [50, 98]
**Total cholesterol, mg/dL**	174 [153, 197]	178 [158, 194]
**LDL cholesterol, mg/dL**	106 [87, 127]	109 [93, 125]
**HDL cholesterol, mg/dL**	52 [44, 61]	50 [43, 55]
**Socioeconomic position at the last attended CARDIA study visit** ^b^ **, *n* (%)**		
**Maximum educational level attained**		
**>High school**	2855 (57%)	17 (25%)
**≤High school**	2192 (43%)	50 (75%)
**Family income, *n* (%)**		
**≥$25,000**	3648 (72%)	31 (46%)
**<$25,000**	950 (19%)	31 (46%)
**Health insurance, *n* (%)**		
**Not having private insurance^c^**	715 (14%)	28 (42%)
**Private**	3814 (76%)	38 (58%)
**Neighborhood deprivation factor score—tertile 3 refers to the most socially deprived, *n* (%)**		
**Tertile 1 range [−4.07, −0.76]**	1699 (34%)	6 (9%)
**Tertile 2 range [−0.76, 0.23]**	1682 (33%)	22 (33%)
**Tertile 3 range [0.23, 2.82]**	1666 (33%)	39 (58%)

CARDIA, Coronary Artery Risk Development for Young Adults; SBP, systolic blood pressure; DBP, diastolic blood pressure; HR, heart rate; BMI, body mass index; LDL, low-density lipoprotein; HDL, high-density lipoprotein; VA, Veterans Affairs.

a Hypertension hospitalization includes being hospitalized for hypertension with target end-organ damage or no end target organ damage.

b Educational level, family income, and insurance category were assessed at the last attended CARDIA study visit up and including year 30. Neighborhood deprivation was assessed at the last attended CARDIA study visit up to and including year 20.

c Not having private insurance: Medicare or Medicaid or VA or no insurance.

The mean age at hypertension hospitalization was 47 years, with the mean (SD) SBP and DBP at the CARDIA visit before hospitalization being 144.8 (27.1) mmHg and 86.4 (17.1) mmHg, respectively (Table S1). The median [25th, 75th percentile] follow-up time from the CARDIA visit before hospitalization to hypertension hospitalization was 5.0 [2.6, 8.9] years. Overall, 63% of participants with a hypertension hospitalization had uncontrolled BP (≥140/90 mmHg) at the last CARDIA visit before hospitalization. The mean (SD) SBP and DBP on admission were 194.1 (29.3) mmHg and 111.5 (18.2) mmHg, respectively. Among the 67 hospitalized patients, 73% (*n* = 49) had end-organ damage at admission or during that hospital stay, including angina (*n* = 26), stroke (*n* = 10), heart failure (*n* = 8), acute kidney injury (*n* = 1), aortic dissection (*n* = 1), atrial fibrillation (*n* = 1), deep vein thrombosis (*n* = 1), end-stage kidney disease (*n* = 1), hypertensive encephalopathy (*n* = 1), and seizures (*n* = 1). Two participants had more than one type of end-organ damage upon admission and no participants had a myocardial infarction. Characteristics of hospitalized participants by socioeconomic position are shown in Table S2A-D.

### Hypertension hospitalization and socioeconomic position

The incidence rate of hypertension hospitalization was higher among participants with ≤high school vs >high school education, those with a family income <$25,000 vs ≥$25,000, those without vs with private insurance, and those residing in more vs less socially deprived neighborhoods (Table 2). After adjustment for age, sex, race, and study center, the hazard ratio (HR) of hospitalization for hypertension was 3.12 (95% CI, 1.78-5.48) for participants with high school or less education vs more than high school education, 2.43 (95% CI, 1.44-4.11) for participants with a family income of less than $25,000 vs family income $25,000 or greater and 2.58 (95% CI, 1.56-4.28) for those not having private insurance vs those with private insurance. Additionally, the HR for tertiles 2 and 3 vs tertile 1 lowest level of neighborhood deprivation (middle and highest vs lowest level of deprivation) was 2.69 (95% CI, 1.08-6.75), and 3.06 (95% CI, 1.23-7.58), respectively.

**Table 2:** Incidence rates and hazard ratios of hypertension hospitalization by socioeconomic position.^a^

	Number of hypertension hospitalizations^b^	Person time in years	Incidence rate of hypertension hospitalizations/10,000 person years (95% CI)	Hazard Ratio (95% CI)^c^
**Maximum educational level attained**				
**>High school**	17	77,049	2.2 (1.2, 3.3)	1 (reference)
**≤High school**	50	50,975	9.8 (7.1, 12.5)	**3.12 (1.78, 5.48)**
**Family income**				
**≥$25,000**	31	99,677	3.1 (2.0, 4.2)	1 (reference)
**<$25,000**	31	23,573	13.2 (8.5, 17.8)	**2.43 (1.44, 4.11)**
**Health insurance**				
**Private**	38	104,354	3.6 (2.5, 4.8)	1 (reference)
**Not having private insurance^d^**	28	19,032	14.71 (9.3, 20.2)	**2.58 (1.56, 4.28)**
**Neighborhood deprivation factor score—tertile 3 refers to the most socially deprived**				
**Tertile 1 range [−4.07, −0.76]**	6	44,708	1.3 (0.3, 2.4)	1 (reference)
**Tertile 2 range [−0.76, 0.23]**	22	42,518	5.2 (3.0, 7.3)	**2.69 (1.08, 6.75)**
**Tertile 3 range [0.23, 2.82]**	39	40,799	9.6 (6.6, 12.6)	**3.06 (1.23, 7.58)**

CARDIA, Coronary Artery Risk Development for Young Adults; VA, Veterans Affairs.

a Educational level, family income, and insurance category were assessed at the last attended CARDIA study visit up and including year 30. Neighborhood deprivation was assessed at the last attended CARDIA study visit up and including year 20.

b Hypertension hospitalization includes being hospitalized for hypertension with target end-organ damage or no end target organ damage.

c Hazard ratio was adjusted for age, sex, race, and study center.

d Not having private insurance: Medicare or Medicaid or VA or no insurance.

### Uncontrolled BP after hypertension hospitalization by socioeconomic position

**Figure 1:**
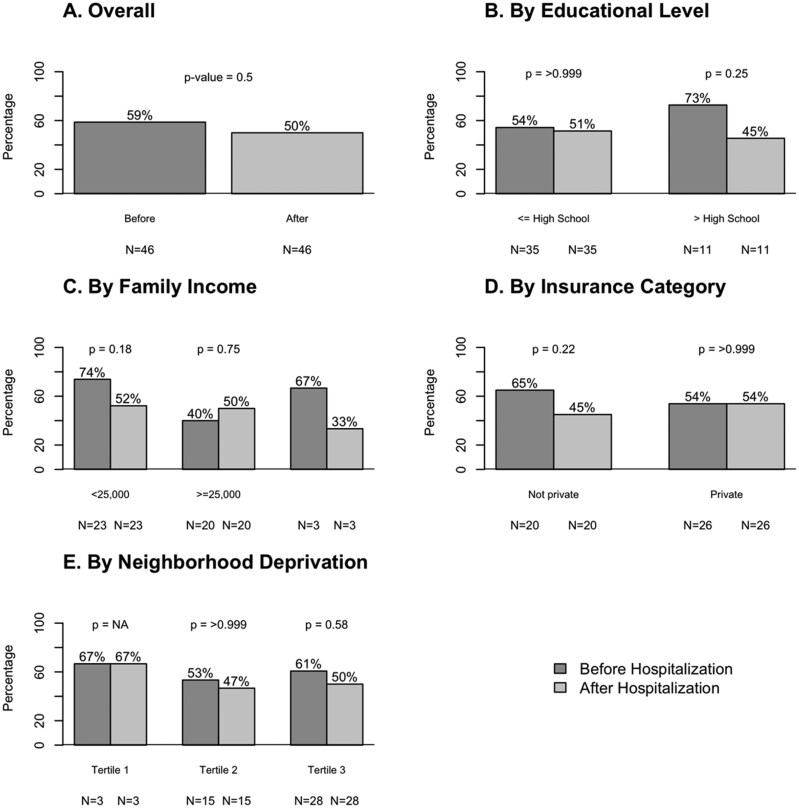
Prevalence of uncontrolled blood pressure (≥140/90 mm Hg) before and after hypertension hospitalization in CARDIA participants. Panel A shows the overall prevalence of uncontrolled blood pressure before and after hospitalization. Panels B–E present stratified analyses by educational level (Panel B), family income (Panel C), insurance category (Panel D), and neighborhood deprivation (Panel E). Data include 46 participants hospitalized for hypertension between the CARDIA baseline visit and the Year 30 examination (2015–2016) who had a subsequent CARDIA follow-up visit. Educational level, family income, and insurance category were assessed at the most recent CARDIA visit up to and including Year 30; neighborhood deprivation was assessed at the most recent CARDIA visit up to and including Year 20. Participants without private insurance included those covered by Medicare, Medicaid, Veterans Affairs, or no insurance. *P*-values compare pre- and post-hospitalization prevalence within strata; *p*-values are not reported (NA) for Panel E due to small sample size.

Of the 67 participants who were hospitalized for hypertension, 8 died before attending another CARDIA study visit, 9 were hospitalized after the final CARDIA exam included in this analysis, and 4 had no contact with the study after hospital discharge. Among the remaining 46 participants who were hospitalized for hypertension, the mean (SD) SBP and DBP were 139.9 (22.4) mmHg and 85.5 (17.6) mmHg, respectively, and 96% of these participants were taking antihypertensive medication at the first CARDIA visit following hospitalization (Table 3). The median time between hypertension hospitalization and the next CARDIA study visit was 3.5 years (25th-75th percentiles: 1.4-5.9 years). Overall, 59% and 50% had SBP ≥140 mmHg or DBP ≥90 mmHg before and after hospitalization, respectively. Figure 1 shows the prevalence of uncontrolled BP before and after hospitalization stratified by socioeconomic position. Using the 2025 ACC/AHA threshold of SBP ≥130 mmHg or DBP ≥80 mmHg, 83% of participants met criteria for uncontrolled BP before hospitalization and 74% after hospitalization. Figure S1 further depicts these differences by socioeconomic position. Socioeconomic position, measured at the participant’s last attended CARDIA visit, was not associated with uncontrolled BP at the subsequent CARDIA examination following hospitalization (Tables S3 and S4).

**Table 3:** Blood pressure, antihypertensive medication use, and blood pressure control following hypertension hospitalization by socioeconomic position.^a^

BP characteristics at first CARDIA visit following hypertension hospitalization	*n*	SBP (95% CI), mmHg	DBP (95% CI), mmHg	Using antihypertensive medication, *n* (%)	Uncontrolled BP (SBP ≥ 140 or DBP ≥ 90 mmHg), *n* (%)	Uncontrolled BP (SBP ≥ 130 or DBP ≥ 80 mmHg), *n* (%)
**Overall**	46	139.9 (22.3)	85.5 (17.6)	44 (96%)	23 (50%)	34 (74%)
**Maximum educational level attained**						
**>High school**	11	139.0 (20.2)	84.1 (14.4)	10 (91%)	5 (45%)	7 (64%)
**≤High school**	35	140.2 (23.3)	85.9 (18.7)	34 (97%)	18 (51%)	27 (77%)
**Family income**						
**≥$25,000**	20	138.3 (15.5)	86.8 (10.4)	19 (95%)	10 (50%)	16 (80%)
**<$25,000**	23	141.6 (27.6)	84.6 (22.4)	23 (100%)	12 (52%)	16 (70%)
**Health insurance**						
**Private**	20	138.3 (23.8)	81.4 (17.6)	25 (96%)	14 (54%)	21 (81%)
**Not having private insurance^b^**	26	141.1 (21.6)	88.7 (17.3)	19 (95%)	9 (45%)	13 (65%)
**Neighborhood deprivation factor score—tertile 3 refers to the most socially deprived**						
**Tertile 1 range [−4.07, −0.76]**	3	157 (30.6)	102.9 (35.1)	3 (100%)	2 (67%)	3 (100%)
**Tertile 2 range [−0.76, 0.23]**	15	135.2 (20.4)	83.7 (11.8)	15 (100%)	7 (47%)	9 (60%)
**Tertile 3 range [0.23, 2.82]**	28	140.5 (22.4)	84.7 (17.8)	26 (93%)	14 (50%)	22 (79%)

CARDIA, Coronary Artery Risk Development in Young Adults; BP, blood pressure; SBP, systolic blood pressure; DBP, diastolic blood pressure; VA, veterans affairs.

a Educational level, family income, and insurance category were assessed at the last attended CARDIA study visit up and including year 30. Neighborhood deprivation was assessed at the last attended CARDIA study visit up and including year 20.

b Not having private insurance: Medicare or Medicaid or VA or no insurance.

A total of 46 participants were hospitalized for hypertension between the CARDIA baseline visit (1985-1986) and year 30 exam (2015-2016) and had a subsequent CARDIA exam.

*P*-value for:.

**• SBP**: Education (.787), Income (.681), Insurance (.364), Neighborhood deprivation (.228).

**• DBP**: Education (.738), Income (.597), Insurance (.166), Neighborhood deprivation (.725).

**• Using antihypertensive medication**: Education (.971), Income (.429), Insurance (>.999), Neighborhood deprivation (.511).

**• Uncontrolled BP (SBP ≥ 140 or DBP ≥ 90 mmHg)**: Education (>.999), Income (>.999), Insurance (.766), Neighborhood deprivation (.819).

**• Uncontrolled BP (SBP ≥ 130 or DBP ≥ 80 mmHg):** Education (.620), Income (.878), Insurance (.385), Neighborhood deprivation (.237).

### Exploratory analysis of the association of socioeconomic position and outcomes following hypertension hospitalization

Among those hospitalized for hypertension, 23 of 67 (34%) participants experienced a cardiovascular event or kidney failure following hospital discharge. Median [25th, 75th percentile] follow-up time from hypertension hospitalization to event was 7.2 [3.6, 16.7] years. Incidence of the outcome was higher among participants who had lower maximum educational level attained, lower family income, and greater neighborhood deprivation (Table S5). The age, sex, race-adjusted risk ratio of experiencing a cardiovascular event or kidney failure was 3.93 (95% CI, 1.39-11.05) among participants with less than high school education compared to those with high school or more education. There was no evidence of an association between family income, health insurance, or neighborhood deprivation with risk of a cardiovascular event or kidney failure after age, sex, and race adjustment.

## Discussion

There are several key findings from the current study that have important implications for public health and clinical practice. Participants with lower socioeconomic position were more likely to have a hypertension hospitalization, with those having lower educational attainment, lower family income, non-private or no health insurance, and residing in more socially deprived neighborhoods at higher risk. Despite being hospitalized for hypertension, 50% of the participants had uncontrolled BP at follow-up, irrespective of their socioeconomic position, even while taking antihypertensive medication.

In the current study, most hospitalizations for hypertension involved target end-organ damage, which may have influenced the decision to admit these patients for inpatient management. An analysis of 33,727 emergency department visits from the 2016 Nationwide Emergency Department Sample found that people who lived in low-income areas and presented to the emergency department for hypertension with and without end-organ damage were more likely to be admitted for inpatient management.^10^ The authors suggested that patients with lower income might lack appropriate outpatient care and, as a result, face a higher risk of cardiovascular events, thus increasing their likelihood of hospital admission.^10^,^27^ Furthermore, a study of Medicare fee-for-service beneficiaries from 1999 to 2019 noted that hospitalization rates for acute hypertension were highest among Black adults and were most prevalent in the southern United States.^1^ These observations are consistent with results of the current study in which most hospitalized patients for hypertension were Black, with a majority from the Birmingham, Alabama CARDIA site. Additionally, approximately 50% of participants hospitalized for hypertension in the current study had uncontrolled hypertension post-discharge, regardless of their socioeconomic position. This represents a modest improvement compared with the 59% who had uncontrolled BP prior to hospitalization.

The findings from the current analysis align with prior research showing that persistent BP elevation is common following hospital discharge for acute hypertension. A study at the Cleveland Clinic Health System of 15,303 adults who were treated for acute BP elevation during non-cardiac hospital stays found that approximately 80% had at least one outpatient systolic BP >139 mmHg or diastolic BP >89 mmHg within a year of discharge.^28^ Many participants had uncontrolled BP following hospital discharge. High-quality post-discharge care for patients with hypertension including establishing a follow-up appointment with primary care clinicians and patient education can improve BP control post-discharge.^18^,^22^ Moreover, the high prevalence of uncontrolled BP following hospital discharge in several prior studies raises the question of whether therapeutic inertia, failure of healthcare providers to initiate or intensify therapy at follow-up outpatient visits, contributes to BP elevation following hypertension hospitalization.^29^,^30^

Hospitalization for hypertension presents an opportunity to identify patients who might benefit optimized BP management strategies post-discharge to reduce adverse cardiovascular events. However, there are no specific recommendations for transitioning care from inpatient to outpatient settings after hospitalization for hypertension (eg, timing of follow-up appointments with a primary care physician or patient education strategies).^18^ In contrast, there are specific recommendations for transitioning care from the emergency department including the referral of patients seen in the emergency department to the outpatient setting, with follow-up recommended within 1-2 months for moderately elevated BP and within 1 week for severely elevated BP.^31^ However, most patients after hospitalization do not receive outpatient care within a timely manner.^31^ Data from the 2013-2014 Nationwide Readmissions Database found that 17.8% of patients admitted for hypertension with end-organ damage were re-hospitalized within 30 days, primarily due to heart failure and hypertension complications.^32^ Moreover, patients insured by Medicaid and those residing in lower-income ZIP codes had higher rates of readmission, highlighting socioeconomic disparities in post-discharge care.^32^ This aligns with findings from the current study, where participants with lower socioeconomic status were more likely to experience subsequent cardiovascular events or kidney failure following hospitalization for hypertension. These observations emphasize the need for improved follow-up care and directed interventions to enhance BP control and reduce readmissions, particularly among those with lower socioeconomic position.

Outpatient guideline-based care to improve BP control includes patient education, lifestyle modification, optimized antihypertensive therapy, and home BP monitoring.^18^,^22^ Scheduling a follow-up primary care appointment or scheduling a patient to see another clinical team member ideally involving a case manager has been shown to be helpful in controlling BP.^18^,^33^ The 2025 ACC/AHA BP guideline and the 2024 AHA Scientific Statement on the management of elevated BP in the acute care setting emphasize the importance of a team-based approach and structured transition-of-care process to ensure continuity following hospitalizations.^18^,^22^ This includes coordination with social workers, pharmacists, and community health workers to address barriers such as access to medications, health insurance, and follow-up care.^18^,^22^ The guideline and scientific statement highlight the need for timely post-discharge follow-up, ideally within weeks after hospitalization, as part of a systematic strategy to achieve and maintain BP control. Enhancing health literacy and leveraging telehealth and remote home BP monitoring can improve communication and enable ongoing management for patients facing logistical or resource barriers.^34–39^ These guideline-based strategies if adopted may improve BP control overall and following hypertension hospitalization.^18^

This study has several strengths. The CARDIA cohort provides over 30 years of follow-up data on Black and White adults from four urban regions across the United States, allowing for a long-term evaluation of hypertension outcomes. BP measurements were taken at multiple study visits using standardized protocols. Additionally, the current analysis included individual and neighborhood-level socioeconomic data, enabling the study of the relationship between socioeconomic position and hypertension hospitalizations. However, the study also has limitations. Hospitalizations were identified based on participant self-report during annual contacts or study examinations, with medical records requested for verification when the reported reason (eg, angina, heart bypass, BP out of control) or symptoms (eg, chest pain or shortness of breath) suggested a possible cardiovascular or pulmonary condition. Some events may have been missed, leading to potential differential misclassification across socioeconomic strata; however, we did not have data to evaluate this potential bias. In addition, the relatively small number of hypertension hospitalizations limited statistical power. As a result, we relied on a minimal adjustment set (age, sex, race, and study center) to reduce the risk of model overfitting. This approach prioritizes model stability but limits our ability to determine which demographic, behavioral, or clinical factors correlated with socioeconomic position are driving the observed associations. Moreover, the timing of follow-up visits after hospitalization varied among participants, which may not fully capture sustained BP control. Finally, data on medication adherence were not available, precluding our ability to assess its potential contribution to persistent uncontrolled BP post-discharge.

Hypertension hospitalizations occurred more frequently among individuals from lower socioeconomic positions, including those with lower educational attainment, lower income, without private health insurance, and those residing in more socially deprived neighborhoods. Despite hospitalization for hypertension and taking antihypertensive medication, half of the participants had uncontrolled BP at follow-up, regardless of socioeconomic position. These findings highlight gaps in hypertension management before and after hospitalization and suggest opportunities to improve BP control during the post-discharge period.

## Supplementary Material

hpaf231_Supplementary_Data

## Data Availability

The data underlying this article can be shared on reasonable request to the corresponding author and approval by the CARDIA study.
