# Supplementary material for: Association of Socioeconomic Position with Incident Hypertension Hospitalization and Blood Pressure Control among Participants in the Coronary Artery Risk Development in Young Adults Study
Source: Am J Hypertens. 2025 Nov 26;39(5):687–95. doi: 10.1093/ajh/hpaf231 (PMC13168809; doi:10.1093/ajh/hpaf231)
Supplement: hpaf231_Supplementary_Data [file hpaf231_supplementary_data.zip › USE - SUPPLEMENTAL MATERIALS - AJH - 10.16.2025.docx]

# **SUPPLEMENTAL MATERIALS**

**Supplementary Data**

# **Figure S1. Prevalence of uncontrolled blood pressure (≥ 130/80 mmHg)** **before or after hypertension hospitalization***

**Table S1. Overall hospitalization characteristics of CARDIA participants who had a hypertension hospitalization**

**Table S2A. Characteristics of CARDIA participants who had a hypertension hospitalization by maximum educational level attained***

# **Table S2B. Characteristics of CARDIA participants who had a hypertension hospitalization by family income***

**Table S2C. Characteristics of CARDIA participants who had a hypertension hospitalization by health insurance***

**Table S2D. Characteristics of CARDIA participants who had a hypertension hospitalization by tertile of neighborhood deprivation factor score***

# **Table S3. Association of socioeconomic position with uncontrolled BP (SBP≥ 140 or DBP≥ 90 mmHg) assessed at the next CARDIA visit after hospitalization using Poisson regression with robust standard errors***^†^

# **Table S4. Association of socioeconomic position with uncontrolled BP (SBP≥ 130 or DBP≥ 80 mmHg) at the next CARDIA visit after hospitalization using Poisson regression with robust standard errors***^†^

**Table S5. Incidence rate and risk ratio of cardiovascular events or kidney failure following hypertension hospitalization by socioeconomic position***^†^ **Supplementary Data***Blood Pressure (BP) measurement*At baseline and years 2 through 15, BP was measured using Hawksley random‐zero sphygmomanometer (W.A. Baum Co., Copiague, NY). At year 20, 25 and 30, BP was measured using a standard automated BP measurement monitor (Omron model HEM907XL; Omron Healthcare, Inc, Lake Forest, IL). BP at year 20 were calibrated to random‐0 sphygmomanometer values based on simultaneous readings from both devices using a Y connector on a subset of Coronary Artery Risk Development in Young Adults (CARDIA) participants to ensure no machine bias remains.^1-3^ Standardized protocols; the details are described in *National Heart* *Lung and Blood Institute, Coronary Artery Risk Development in Young Adults (CARDIA) Study Manual of Operation, 1985.^3^* Hypertension was defined as having Systolic BP (DBP) ≥ 140 and Diastolic BP (DBP) ≥ 90 mm Hg or self-report of hypertension with antihypertensive medication treatment.

All participants with uncontrolled BP at the CARDIA study visit were notified, offered to have their BP re-measured and were referred to source of care.

*Neighborhood socioeconomic position*Data for the neighborhood deprivation score were obtained from United States Census data, and **census-defined block groups were used as proxies for participants’ neighborhoods**. A composite z-score for each of these variables was calculated and the sum was used as the neighborhood deprivation score for all the CARDIA cohort.

Neighborhood deprivation score for year 0 was based on census data from 1980 normalized to 2010, for year 7 and 10 it was based on census data from year 1990 normalized to 2010, for year 15 and 20 it was based on census data from year 2000 normalized to year 2010. We did not include data from year 25 or year 30 since they were based on the American Census Survey and not Census data. For each participant we used the data available at the last CARDIA visit attended between year 0 and year 20.

*Covariate assessment at each study visit*Race, sex, smoking history, and alcohol use were self-reported by participants. Body mass index (BMI) was calculated as measured weight in kilograms divided by height in meters squared (kg/m^2^). Hypertension was defined as having SBP ≥ 140 and DBP ≥ 90 mm Hg or self-report of hypertension with antihypertensive medication treatment. A participant was considered to have diabetes either by self-report of diabetes with antidiabetic medication treatment or elevated fasting glucose (126 mg/dL) or 2-hour postload serum glucose from the 75 g oral glucose tolerance tests (200 mg/dL or greater) and/or glycated hemoglobin (6.5% or greater).^4,5^ A participant was considered to have chronic kidney disease (CKD) either by self-report or if they have an estimated glomerular filtration rate (eGFR) ≤ 60 ml/min/1.73 m^2^ according to the 2021 Chronic Kidney Disease Epidemiology Collaboration creatinine equation without race.^6,7^ Detailed description of blood specimen collection and methodologies to assay concentrations of total cholesterol, triglycerides, high‐density lipoprotein cholesterol (HDL), low‐density lipoprotein cholesterol (LDL) were reported elsewhere.^8,9^

*Cardiovascular events, kidney failure events and death*Cardiovascular events, kidney failure events, and death were tracked from the baseline examination through August 31, 2020, with follow-up extending over 34 years. Participants reported hospitalizations and outpatient procedures during in-clinic visits and annual follow-up contacts. Medical records were obtained for self-reported conditions of interest (e.g., heart failure, heart attack) or symptoms that could be due to a condition of interest (e.g. shortness of breath, chest pain).^10-13^ Deaths were identified through attempted annual contact with the participant or a participant-designated proxy, internet searches, and periodic linkage to the National Death Index. For deceased participants, medical and death records were obtained with consent from the next of kin. All recorded events-including cardiovascular events, kidney failure, and deaths-were adjudicated by two physician members of the Outcomes Surveillance and Adjudication Subcommittee, with discrepancies resolved by the full committee. Incident cardiovascular events included nonfatal myocardial infarction or stroke; hospitalization for acute coronary syndrome not resulting in myocardial infarction, congestive heart failure, or transient ischemic attack; revascularization procedures or evidence of carotid artery obstruction or peripheral arterial disease based on angiographic or ultrasonographic findings; and deaths due to coronary heart disease, stroke, atherosclerotic disease (excluding coronary or stroke-related cases), or nonatherosclerotic cardiac conditions (e.g. cardiomyopathy). Incident kidney failure was identified based on initiation of chronic dialysis, or the need for or actual kidney transplantation. Participants who did not experience an event and remained in the study were censored as of August 31, 2020.

*References:*
1. National Heart Lung and Blood Institute. Coronary Artery Risk Develop ment in Young Adults (CARDIA) study manuals of operation. 1985. [http://www.cardia.dopm.uab.edu/exam-materials2/manual-of-operations. Accessed October 2025](http://www.cardia.dopm.uab.edu/exam-materials2/manual-of-operations.%20Accessed%20October%202025).

2. Friedman GD, Cutter GR, Donahue RP, et al. CARDIA: study design, recruitment, and some characteristics of the examined subjects. J Clin Epidemiol. 1988;41:1105-1116.
3. Jacobs DR Jr, Yatsuya H, Hearst MO, et al. Rate of decline of forced vital capacity predicts future arterial hypertension: the Coronary Artery Risk Development in Young Adults Study. *Hypertension*. 2012;59(2):219-225. doi:10.1161/HYPERTENSIONAHA.111.184101
2. American Diabetes Association. Diagnosis and classification of diabetes mellitus. *Diabetes Care*. 2014;37 Suppl 1:S81-S90. doi:10.2337/dc14-S081
3. Gunderson EP, Lewis CE, Lin Y, et al. Lactation Duration and Progression to Diabetes in Women Across the Childbearing Years: The 30-Year CARDIA Study. *JAMA Intern Med*. 2018;178(3):328-337. doi:10.1001/jamainternmed.2017.7978
4. Levey AS, Eckardt KU, Dorman NM, et al. Nomenclature for Kidney Function and Disease: Executive Summary and Glossary From a Kidney Disease: Improving Global Outcomes (KDIGO) Consensus Conference. *Am J Kidney Dis*. 2020;76(2):157-160. doi:10.1053/j.ajkd.2020.05.005
5. Delgado C, Baweja M, Crews DC, et al. A Unifying Approach for GFR Estimation: Recommendations of the NKF-ASN Task Force on Reassessing the Inclusion of Race in Diagnosing Kidney Disease. *Am J Kidney Dis*. 2022;79(2):268-288.e1. doi:10.1053/j.ajkd.2021.08.003
6. Friedewald WT, Levy RI, Fredrickson DS. Estimation of the concentration of low-density lipoprotein cholesterol in plasma, without use of the preparative ultracentrifuge. *Clin Chem*. 1972;18(6):499-502.
7. Warnick GR, Benderson J, Albers JJ. Dextran sulfate-Mg2+ precipitation procedure for quantitation of high-density-lipoprotein cholesterol. *Clin Chem*. 1982;28(6):1379-1388.

8. Adams H.P., Jr., Bendixen B.H., Kappelle L.J., Biller J., Love B.B., Gordon D.L., et al. Classification of subtype of acute ischemic stroke definitions for use in a multicenter clinical trial. TOAST. Trial of Org 10172 in Acute Stroke Treatment, Stroke. 1993;24(1):35–41.

9. Easton J.D., Saver J.L., Albers G.W., Alberts M.J., Chaturvedi S., Feldmann E., et al. Definition and evaluation of transient ischemic attack: a scientific statement for healthcare professionals from the American Heart Association/American Stroke Association Stroke Council; Council on Cardiovascular Surgery and Anesthesia; Council on Cardiovascular Radiology and Intervention; Council on Cardiovascular Nursing; and the Interdisciplinary Council on Peripheral Vascular Disease. Stroke. 2009;40(6):2276–2293.

10. Luepker R.V., Apple F.S., Christenson R.H., Crow R.S., Fortmann S.P., Goff D., et al. Case definitions for acute coronary heart disease in epidemiology and clinical research studies: a statement from the AHA Council on Epidemiology and Prevention; the European Society of Cardiology Working Group on Epidemiology and Prevention; Centers for Disease Control and Prevention; and the National Heart, Lung, and Blood Institute. Circulation. 2003;108(20):2543–2549.

11. Madden K.P., Karanjia P.N., Adams H.P., Jr., Clarke W.R. Accuracy of initial stroke subtype diagnosis in the TOAST study. Trial of ORG 10172 in Acute Stroke Treatment. Neurology. 1995;45(11):1975–1999.
12. Rosamond W.D., Chang P.P., Baggett C., Johnson A., Bertoni A.G., Shahar E., et al. Classification of heart failure in the atherosclerosis risk in communities (ARIC) study: a comparison of diagnostic criteria, Circ. Heart Fail. 2012;5(2):152–159. 
13. Thygesen K., Alpert J.S., Jaffe A.S., Simoons M.L., Chaitman B.R., White H.D., et al. Third universal definition of myocardial infarction. Glob. Heart. 2012;7(4):275–295.

# **Figure S1. Prevalence of uncontrolled blood pressure (≥ 130/80 mmHg)** **before or after hypertension hospitalization***

# **
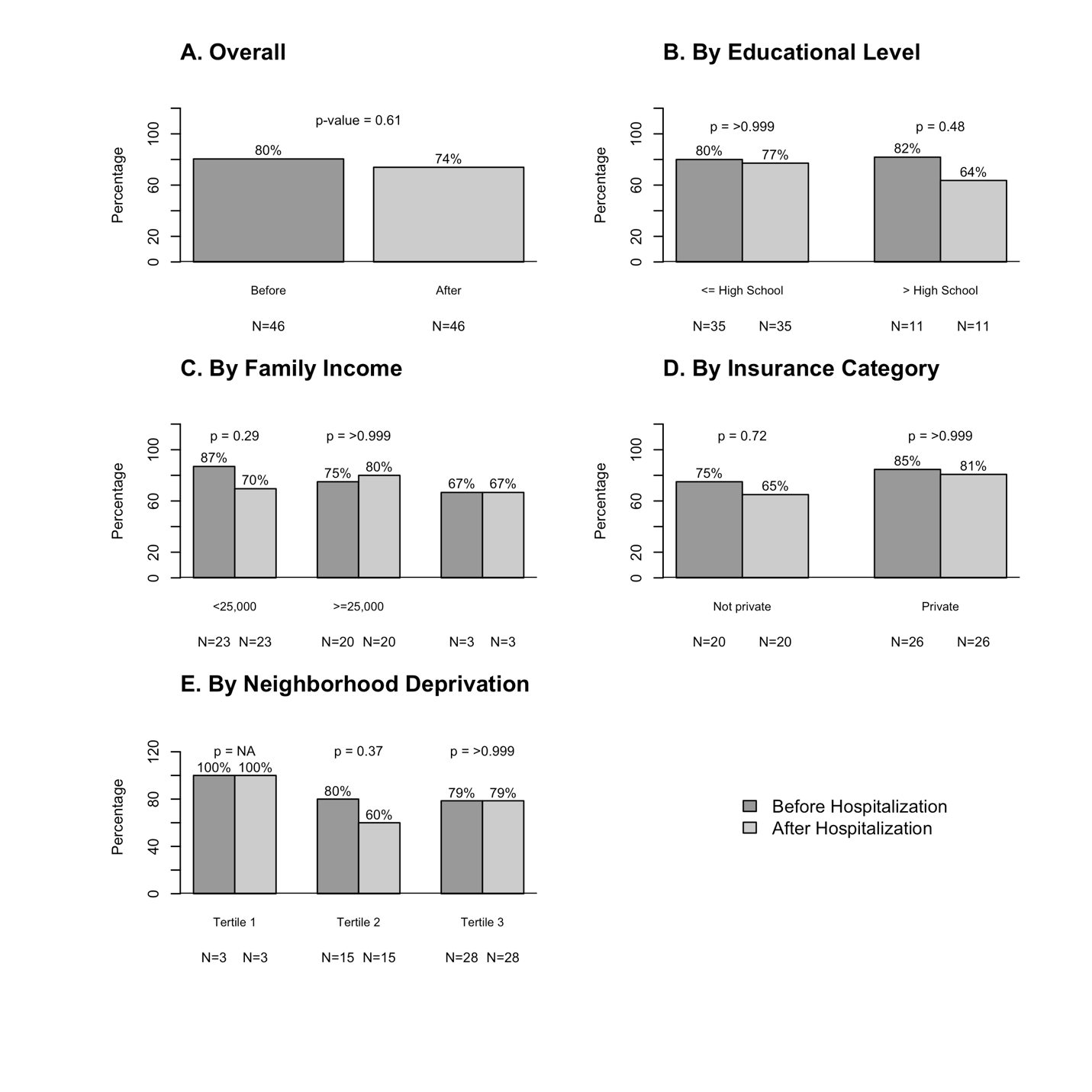
**

CARDIA: Coronary Artery Risk Development for Young Adults

The figure includes participants (n = 46) who were hospitalized for hypertension and had blood pressure measured at both a CARDIA visit before and after hospitalization.

Percentages indicate the proportion of participants with uncontrolled blood pressure.
**N**values beneath the bars represent the total number of participants in each subgroup.

Educational level, family income, and insurance category were assessed at the last attended CARDIA visit through year 30; neighborhood deprivation was assessed through year 20. Not having private insurance refers to participants covered by Medicare, Medicaid, Veterans Affairs, or without insurance. p-value is not assessed (NA) in Panel E due to the small sample size.

**Table S1. Overall hospitalization characteristics of CARDIA participants who had a hypertension hospitalization**

|  | Participants who had a hypertension hospitalization N= 67 |
| --- | --- |
| **BP characteristics at visit immediately before hypertension hospitalization** | |
| SBP, mmHg, mean (SD) | 144.8 (27.1) |
| DBP, mmHg, mean (SD) | 86.4 (17.1) |
| Using antihypertensive medications, n (%) | 27 (44%) |
| BP uncontrolled (SBP≥140 and DBP≥90 mmHg), n (%) | 42 (63%) |
| BP uncontrolled (SBP≥130 and DBP≥80 mmHg), n (%) | 54 (81%) |
| **Age on admission**, mean (SD) | 47 (8) |
| **BP on admission (from discharge note),** | |
| SBP, mmHg, mean (SD) | 194.1 (29.3) |
| DBP, mmHg, mean (SD) | 111.5 (18.2) |
| **Had target end organ damage,** n (%) | 49 (73%) |
| **BP on discharge (from discharge note)** | |
| SBP, mmHg, mean (SD) | 142.5 (21.1) missing in n=49 |
| DBP, mmHg, mean (SD) | 78.6 (12.2) |
| **Prescribed antihypertensives at discharge (from discharge note),** n (%) | 53/55 (96%) missing in n=12 |
| **Length of stay, days,** median [25^th^ percentile, 75^th^ percentile] | 2 [1, 5] |

SBP: systolic blood pressure; DBP: diastolic blood pressure, BP: blood pressure; SD: standard deviation

**Table S2A. Characteristics of CARDIA participants who had a hypertension hospitalization by maximum educational level attained***

|  | ≤ High school  N= 50 (75%) | > High school N= 17 (25%) | p-values |
| --- | --- | --- | --- |
| **Demographics** | | | |
| Age at time of hypertension hospitalization, mean (SD) | 45 (8) | 46 (3) | 0.784 |
| Male sex at baseline, n (%) | 26 (52%) | 8 (47%) | 0.943 |
| Black race at baseline, n (%) | 44 (88%) | 14 (82%) | 0.859 |
| **Study center at baseline**, n (%) | | | |
| Birmingham | 25 (50%) | 8 (47%) | 0.179 |
| Chicago | 13 (36%) | 2 (12%) |  |
| Minnesota | 3 (6%) | 4 (24%) |  |
| Oakland | 9 (18%) | 3 (18%) |  |
| **Heath Behaviors at visit immediately before hospitalization**, n (%) | | | |
| **Smoking status** | | | |
| Never smoker | 20 (40%) | 10 (59%) | 0.264 |
| Former smoker | 4 (8%) | 0 (0%) |  |
| Current smoker | 26 (52%) | 7 (41%) |  |
| **Drank alcohol in the past year** | 32 (64%) | 10 (59%) | 0.928 |
| **Medical history at visit immediately before hospitalization**, n (%) | | | |
| Hypertension | 30 (60%) | 10 (59%) | >0.999 |
| Diabetes | 8 (16%) | 4 (24%) | 0.739 |
| Chronic Kidney Disease | 10 (20%) | 4 (24%) | >0.999 |
| **BMI and laboratory values at visit immediately before hospitalization**, median [25^th^ percentile, 75^th^ percentile] | | | |
| BMI, kg/m^2^ | 32.4 [27.4, 37.1] | 31.9 [28.9, 35.8] | 0.851 |
| Triglycerides, mg/dL | 101 [79, 128] | 89 [74, 112] | 0.428 |
| Total cholesterol, mg/dL | 183 [162, 202] | 194 [127, 219] | 0.746 |
| LDL cholesterol, mg/dL | 106 [85, 124] | 107 [66, 130] | 0.757 |
| HDL cholesterol, mg/dL | 49 [42, 58] | 55 [46, 71] | 0.207 |
| **BP characteristics at visit immediately before hospitalization** | | | |
| SBP, mmHg, mean (SD) | 143.4 (27.5) | 148.7 (26.2) | 0.497 |
| DBP, mmHg, mean (SD) | 85.2 (15.7) | 89.9 (20.7) | 0.332 |
| Using antihypertensive medications, n (%) | 21 (42%) | 6 (35%) | 0.784 |
| BP uncontrolled (SBP≥140 and DBP≥90 mmHg), n (%) | 30 (60%) | 12 (71%) | 0.624 |
| BP uncontrolled (SBP≥130 and DBP≥80 mmHg), n (%) | 40 (80%) | 14 (82%) | >0.999 |
| **Hypertension hospitalization characteristics** | | | |
| **BP on admission (from discharge note)** | | | |
| SBP, mmHg, mean (SD) | 193.9 (29.2) | 194.7 (30.6) | 0.929 |
| DBP, mmHg, mean (SD) | 110.1 (17.5) | 115.8 (19.9) | 0.267 |
| **Had target end organ damage,** n (%) | 36 (72%) | 13 (77%) | 0.966 |
| **BP on discharge (from discharge note)** | | | |
| SBP, mmHg, mean (SD) | 139.9 (22.5) missing in n=35 | 151.5 (13.9)  missing in n= 13 | 0.348 |
| DBP, mmHg, mean (SD) | 77.6 (12.9) missing in n= 35 | 82.0 (10.2) missing in n= 13 | 0.538 |
| **Prescribed antihypertensives at discharge (from discharge note)**, n (%) | 38/39 (97%) missing in n= 35 | 15/16 (94%) missing in n= 25 | 0.257 |
| **Length of stay, days**, median [25^th^ percentile, 75^th^ percentile] | 2 [1, 5] | 3 [1, 5] | 0.656 |

Continuous variables were compared using Student’s t-test or Wilcoxon rank-sum test, as appropriate. Categorical variables were compared using Pearson’s Chi-squared test or Fisher’s exact test for small cell counts.

SD: standard deviation; SBP: systolic blood pressure; DBP: diastolic blood pressure; HR: heart rate; BMI: body mass index; LDL: low density lipoprotein; HDL: high density lipoprotein; VA: Veterans Affairs

* Maximum educational level assessed at the participant's last attended CARDIA study visit up and including year 30

- Among participants with ≤ high school education, end organ damage included: acute kidney injury (n=1), heart failure (n=5), stroke (n=7), angina (n=19), deep vein thrombosis (n=1), Atrial fibrillation (n=1), aortic dissection (n=1), hypertensive encephalopathy (n=1), end stage renal disease (n=1), and seizure (n=1). Two participants had more than 1 condition.
- Among participants with > high school education, end organ damage included: heart failure (n=3), stroke (n=3), and angina (n=7).

# **Table S2B. Characteristics of CARDIA participants who had a hypertension hospitalization by family income***

|  | < $25,000 N= 31 (48%) | ≥ $25,000 N= 31 (52%) | p-values |
| --- | --- | --- | --- |
| **Demographics** | | | |
| Age at time of hypertension hospitalization, mean (SD) | 46 (9) | 47 (8) | 0.391 |
| Male sex at baseline, n (%) | 15 (48%) | 16 (52%) | >0.999 |
| Black race at baseline, n (%) | 29 (94%) | 25 (81%) | 0.256 |
| **Study center at baseline**, n (%) | | | |
| Birmingham | 18 (58%) | 11 (36%) | 0.230 |
| Chicago | 5 (16%) | 9 (29%) |  |
| Minnesota | 4 (13%) | 3 (10%) |  |
| Oakland | 4 (13%) | 8 (26%) |  |
| **Heath Behaviors at visit immediately before hospitalization**, n (%) | | | |
| **Smoking status** | | | |
| Never smoker | 10 (32%) | 19 (61%) | 0.064 |
| Former smoker | 3 (10%) | 1 (3%) |  |
| Current smoker | 18 (58%) | 11 (36%) |  |
| **Drank alcohol in the past year** | 17 (55%) | 23 (74%) | 0.184 |
| **Medical history at visit immediately before hospitalization**, n (%) | | | |
| Hypertension | 19 (61%) | 19 (61%) | >0.999 |
| Diabetes | 6 (19%) | 5 (16%) | >0.999 |
| Chronic Kidney Disease | 6 (19%) | 7 (23%) | >0.999 |
| **BMI and laboratory values at visit immediately before hospitalization**, median [25^th^ percentile, 75^th^ percentile] | | | |
| BMI, kg/m^2^ | 32.4 [30.0, 36.2] | 31.9 [27.1, 38.5] | 0.849 |
| Triglycerides, mg/dL | 102 [80, 133] | 96 [71, 124] | 0.602 |
| Total cholesterol, mg/dL | 185 [156, 213] | 183 [167, 201] | 0.888 |
| LDL cholesterol, mg/dL | 104 [81, 125] | 113 [83, 132] | 0.536 |
| HDL cholesterol, mg/dL | 52 [43, 63] | 50 [39, 59] | 0.263 |
| **BP characteristics at visit immediately before hospitalization** | | | |
| SBP, mmHg, mean (SD) | 149.3 (25.7) | 142.3 (24.5) | 0.275 |
| DBP, mmHg, mean (SD) | 88.6.2 (15.2) | 84.4 (17.0) | 0.314 |
| Using antihypertensive medications, n (%) | 9 (29%) | 16 (52%) | 0.044 |
| BP uncontrolled (SBP≥140 and DBP≥90 mmHg), n (%) | 23 (74%) | 17 (55%) | 0.184 |
| BP uncontrolled (SBP≥130 and DBP≥80 mmHg), n (%) | 28 (90%) | 24 (77%) | 0.300 |
| **Hypertension hospitalization characteristics** | | | |
| **BP on admission (from discharge note)** | | | |
| SBP, mmHg, mean (SD) | 190.6 (24.7) | 194.8 (31.8) | 0.555 |
| DBP, mmHg, mean (SD) | 108.9 (16.4) | 113.0 (19.1) | 0.371 |
| **Had target end organ damage**, n (%) | 20 (65%) | 25 (81%) | 0.255 |
| **Vitals on discharge (from discharge note)** | | | |
| SBP, mmHg, mean (SD) | 152.9 (22.3) missing in n= 22 | 135.5 (17.3) missing in n= 22 | 0.090 |
| DBP, mmHg, mean (SD) | 81.8 (12.4) missing in n= 22 | 75.0 (12.1) missing in n= 22 | 0.250 |
| **Prescribed antihypertensives at discharge (from discharge note)**, n (%) | 23/23 (100%) missing in n=8 | 26/27 (96%) missing in n=4 | 0.284 |
| **Length of stay, days**, median [25^th^ percentile, 75^th^ percentile] | 2 [1, 5] | 3 [1, 4] | 0.276 |

Continuous variables were compared using Student’s t-test or Wilcoxon rank-sum test, as appropriate. Categorical variables were compared using Pearson’s Chi-squared test or Fisher’s exact test for small cell counts.

SD: standard deviation; SBP: systolic blood pressure; DBP: diastolic blood pressure; HR: heart rate; BMI: body mass index; LDL: low density lipoprotein; HDL: high density lipoprotein; VA: Veterans Affairs

* Family income assessed at the participant's last attended CARDIA study visit up and including year 30

- 5 participants had missing data on family income.
- Among participants with family income < $25,000, end organ damage included: heart failure (n=3), stroke (n=3), angina (n=11), aortic dissection (n=1), end stage renal disease (n=1), and seizure (n=1). One participant had 2 conditions.
- Among participants with family income ≥ $25,000, end organ damage included: acute kidney injury (n=1), heart failure (n=4), stroke (n=4), angina (n=14), deep vein thrombosis (n=1), atrial fibrillation (n=1), and hypertensive encephalopathy (n=1). One participant had 2 conditions.

**Table S2C. Characteristics of CARDIA participants who had a hypertension hospitalization by health insurance***

|  | Not having private insurance^1^ N= 28 (42%) | Private  N=38 (58%) | p-values |
| --- | --- | --- | --- |
| **Demographics** | | | |
| Age at time of hypertension hospitalization, mean ± SD | 47 (8) | 46 (9) | 0.790 |
| Male sex at baseline (%) | 15 (54%) | 19 (50%) | 0.970 |
| Black race at baseline, n (%) | 25 (89%) | 33 (87%) | >0.999 |
| **Study center at baseline**, n (%) | | | |
| Birmingham | 14 (50%) | 18 (47%) | 0.999 |
| Chicago | 7 (25%) | 8 (21%) |  |
| Minnesota | 3 (11%) | 4 (11%) |  |
| Oakland | 4 (14%) | 8 (21%) |  |
| **Heath Behaviors at visit immediately before hospitalization**, n (%) | | | |
| **Smoking status** | | | |
| Never smoker | 10 (36%) | 20 (53%) | 0.394 |
| Former smoker | 2 (7%) | 2 (5%) |  |
| Current smoker | 16 (57%) | 16 (42%) |  |
| **Drank alcohol in the past year** | 16 (57%) | 26 (68%) | 0.495 |
| **Medical history at visit immediately before hospitalization**, n (%) | | | |
| Hypertension | 18 (64%) | 22 (58%) | 0.787 |
| Diabetes | 5 (18%) | 7 (18%) | >0.999 |
| Chronic Kidney Disease | 4 (14%) | 10 (26%) | 0.381 |
| **BMI and laboratory values at visit immediately before hospitalization**, median [25^th^ percentile, 75^th^ percentile] | | | |
| BMI, kg/m^2^ | 32.0 [29.5, 35.1] | 32.5 [27.0, 37.7] | 0.765 |
| Triglycerides, mg/dL | 108 [80, 153] | 96 [69, 120] | 0.347 |
| Total cholesterol, mg/dL | 184 [162, 204] | 183 [155, 208] | 0.613 |
| LDL cholesterol, mg/dL | 102 [82, 125] | 113 [82, 129] | 0.678 |
| HDL cholesterol, mg/dL | 55 [47, 63] | 47 [38, 57] | 0.026 |
| **BP characteristics at visit immediately before hospitalization** | | | |
| SBP, mmHg, mean (SD) | 153.1 (27.3) | 140.3 (24.2) | 0.049 |
| DBP, mmHg, mean (SD) | 88.4 (17.7) | 85.8 (16.2) | 0.548 |
| Using antihypertensive medications, n (%) | 12 (43%) | 23 (61%) | 0.363 |
| BP uncontrolled (SBP≥140 and DBP≥90 mmHg), n (%) | 20 (71%) | 22 (58%) | 0.384 |
| BP uncontrolled (SBP≥130 and DBP≥80 mmHg), n (%) | 23 (82%) | 31 (82%) | >0.999 |
| **Hypertension hospitalization characteristics** | | | |
| **BP on admission (from discharge note)** | | | |
| SBP, mmHg, mean (SD) | 199.6 (29.4) | 189.8 (29.4) | 0.186 |
| DBP, mmHg, mean (SD) | 112.3 (17.9) | 111.3 (18.7) | 0833 |
| **Had target end organ damage,** n (%) | 19 (68%) | 29 (76%) | 0.629 |
| **BP on discharge (from discharge note)** | | | |
| SBP, mmHg, mean (SD) | 143.8 (23.6) missing in n=20 | 141.5 (20.1) missing in n=28 | 0.830 |
| DBP, mmHg, mean (SD) | 78.3 (10.7) missing in n=20 | 78.8 (13.8)  missing in n=28 | 0.928 |
| **Prescribed antihypertensives at discharge (from discharge note)**, n (%) | 23/24 (96%) missing in n=4 | 29/30 (97%) missing in n=8 | 0.770 |
| **Length of stay, days**, median [25^th^ percentile, 75^th^ percentile] | 2 [1, 5] | 1 [1, 5] | 0.506 |

Continuous variables were compared using Student’s t-test or Wilcoxon rank-sum test, as appropriate. Categorical variables were compared using Pearson’s Chi-squared test or Fisher’s exact test for small cell counts.

SD: standard deviation; SBP: systolic blood pressure; DBP: diastolic blood pressure; HR: heart rate; BMI: body mass index; LDL: low density lipoprotein; HDL: high density lipoprotein; VA: Veterans Affairs

* Health insurance (not having private insurance [Medicare or Medicaid or VA or no insurance] versus no insurance) assessed at the participant's last attended CARDIA study visit up and including year 30

- 1 participant has missing data on insurance at last study visit.
- Among participants with Medicare or Medicaid or no insurance or VA, end organ damage included: heart failure (n=6), stroke (n=4), angina (n=8), aortic dissection (n=1), hypertensive encephalopathy (n=1), and seizure (n=1). Two participants had 2 conditions.
- Among participants with private insurance, end organ damage included: acute kidney injury (n=1), heart failure (n=2), stroke (n=6), angina (n=17), deep vein thrombosis (n=1), atrial fibrillation (n=1), and end stage renal disease (n=1).

**Table S2D. Characteristics of CARDIA participants who had a hypertension hospitalization by tertile of neighborhood deprivation factor score***

|  | Tertile 1 range  [-4.07, -0.76) N=6 (9%) | Tertile 2 range  [-0.76, 0.23) N=22 (33%) | Tertile 3 range [0.23, 2.82] N=39 (58%) | p-values |
| --- | --- | --- | --- | --- |
| **Demographics** | | | | |
| Age at time of hypertension hospitalization, mean (SD) | 45 (8) | 46 (3) | 46 (5) | 0.784 |
| Male sex at baseline (%) | 3 (50%) | 9 (41%) | 22 (56%) | 0.247 |
| Black race at baseline, n (%) | 5 (83%) | 17 (77%) | 36 (92%) |  |
| **Study center at baseline**, n (%) | | | | |
| Birmingham | 0 (0%) | 9 (41%) | 24 (62%) | 0.003 |
| Chicago | 1 (17%) | 4 (18%) | 10 (26%) |  |
| Minnesota | 1 (17%) | 5 (23%) | 1 (3%) |  |
| Oakland | 4 (67%) | 4 (18%) | 4 (10%) |  |
| **Heath Behaviors at visit immediately before hospitalization**, n (%) | | | | |
| **Smoking status** | | | | |
| Never smoker | 4 (67%) | 13 (59%) | 13 (33%) | 0.099 |
| Former smoker | 1 (17%) | 0 (0%) | 3 (8%) |  |
| Current smoker | 1 (17%) | 9 (41%) | 23 (59%) |  |
| **Drank alcohol in the past year** | 5 (83%) | 14 (64^) | 23 (59%) | 0.514 |
| **Medical history at visit immediately before hospitalization**, n (%) | | | | |
| Hypertension | 3 (50%) | 12 (55%) | 25 (64%) | 0.673 |
| Diabetes | 0 (0%) | 6 (27%) | 6 (15%) | 0.248 |
| Chronic Kidney Disease | 1 (17%) | 6 (27%) | 7 (18%) | 0.667 |
| **BMI and laboratory values at visit immediately before hospitalization**, median [25^th^ percentile, 75^th^ percentile] | | | | |
| BMI, kg/m^2^ | 33.5 [29.9, 37.2] | 32.7 [30.1, 40.7] | 31.9 [26.9, 34.4] | 0.375 |
| Triglycerides, mg/dL | 90 [72, 101] | 106 [70, 217] | 96 [80, 195] | 0.481 |
| Total cholesterol, mg/dL | 189 [174, 211] | 196 [170, 218] | 179 [161, 195] | 0.419 |
| LDL cholesterol, mg/dL | 128 [93, 143] | 110 [67, 134] | 102 [85, 122] | 0.494 |
| HDL cholesterol, mg/dL | 46 [43, 48] | 49 [39, 60] | 52 [46, 62] | 0.480 |
| **BP characteristics at visit immediately before hospitalization** | | | | |
| SBP, mmHg, mean (SD) | 147.1 (24.0) | 141.4 (30.9) | 146.3 (25.7) | 0.778 |
| DBP, mmHg, mean (SD) | 91.6 (18.9) | 84.1 (17.2) | 86.9 (16.9) | 0.616 |
| Using antihypertensive medications, n (%) | 4 (67%) | 10 (46%) | 13 (33%) | 0.355 |
| BP uncontrolled (SBP≥140 and DBP≥90 mmHg), n (%) | 4 (67%) | 12 (55%) | 26 (67%) | 0.629 |
| BP uncontrolled (SBP≥130 and DBP≥80 mmHg), n (%) | 5 (83%) | 17 (77%) | 32 (82%) | 0.888 |
| **Hypertension hospitalization characteristics** | | | | |
| **BP on admission (from discharge note)** | | | | |
| SBP, mmHg, mean (SD) | 202.7 (20.0) | 193.4 (28.8) | 193.2 (31.2) | 0.760 |
| DBP, mmHg, mean (SD) | 118.8 (29.1) | 110.9 (17.1) | 110.7 (17.1) | 0.592 |
| **Had target end organ damage**, n (%) | 4 (67%) | 20 (91%) | 25 (64%) | 0.071 |
| **Vitals on discharge (from discharge note)** | | | | |
| SBP, mmHg, mean (SD) | 134 (NA) missing in n=5 | 139.3 (20.6) missing in n=18 | 144.2 (22.6) missing in n=25 | 0.899 |
| DBP, mmHg, mean (SD) | 91.0 (NA) missing in n=5 | 75.8 (12.3) missing in n=18 | 78.5 (12.5) missing in n=25 | 0.424 |
| **Prescribed antihypertensives at discharge (from discharge note)**, n (%) | 4/4 (100%) missing in n=2 | 17/18 (94%) missing in n=4 | 32/33 (97%) missing in n=6 | 0.829 |
| **Length of stay, days**, median [25^th^ percentile, 75^th^ percentile] | 5 [1, 6] | 2 [1, 4] | 2 [1, 5] | 0.830 |

One-way ANOVA was used for continuous variables and Pearson’s Chi-squared test or Fisher’s exact test for categorical variables, as appropriate

SD: standard deviation; SBP: systolic blood pressure; DBP: diastolic blood pressure; HR: heart rate; BMI: body mass index; LDL: low density lipoprotein; HDL: high density lipoprotein; VA: Veterans Affairs

* Neighborhood deprivation factor score assessed at the participant's last attended CARDIA study visit up and including year 20

- Among participants within tertile 1 of neighborhood deprivation factor score, end organ damage included: acute kidney injury (n=1), heart failure (n=1), stroke (n=1), angina (n=1),
- Among participants within tertile 2 of neighborhood deprivation factor score, end organ damage included: heart failure (n=3), stroke (n=4), angina (n=13), deep vein thrombosis (n=1), and atrial fibrillation (n=1). Two participants had 2 conditions.
- Among participants within tertile 3 of neighborhood deprivation factor score, end organ damage included: heart failure (n=4), stroke (n=5), angina (n=12), aortic dissection (n=1), hypertensive encephalopathy (n=1), end stage renal disease (n=1) and seizure (n=1).

# **Table S3. Association of socioeconomic position with uncontrolled BP (SBP≥ 140 or DBP≥ 90 mmHg) assessed at the next CARDIA visit after hospitalization using Poisson regression with robust standard errors***^†^

| Risk Ratio (95% CI) | Model 1 | Model 2 |
| --- | --- | --- |
| **Maximum educational level attained** | | |
| > High school | Reference | Reference |
| ≤ High school | 1.00 [0.46, 2.20] | 1.17 [0.53, 2.56] |
| **Family income** | | |
| ≥ $25,000 | Reference | Reference |
| < $25,000 | 1.05 [0.59, 1.85] | 0.81 [0.45, 1.47] |
| **Health insurance** | | |
| Private | Reference | Reference |
| Not having private insurance ^‡^ | 1.21 [0.67, 2.18] | 1.10 [0.63, 1.94] |
| **Neighborhood deprivation factor score – tertile 3 refers to the most socially deprived** | | |
| Tertile 1 range  [-4.07, -0.76) | Reference | Reference |
| Tertile 2 range  [-0.76, 0.23) | 0.66 [0.20, 2.13] | 0.49 [0.17, 1.39] |
| Tertile 3 range  [0.23, 2.82] | 0.68 [0.23, 2.01] | 0.71 [0.22, 2.24] |

BP: blood pressure; SBP: systolic blood pressure; DBP: diastolic blood pressure; CARDIA: Coronary Artery Risk Development in Young Adults; VA: Veterans Affairs

* This includes 46 participants who were hospitalized for hypertension between the CARDIA baseline visit and year 30 exam (2015–2016) and who had a subsequent CARDIA exam.

† Educational level, family income and insurance category were assessed at the last attended CARDIA study visit up and including year 30. Neighborhood deprivation was assessed at the last attended CARDIA study visit up and including year 20.

‡ Not having private insurance: Medicare or Medicaid or VA or no insurance

Model 1: adjust for age, sex, race, center (age at time of hypertension hospitalization, sex and center from Y0)
Model 2: Model 1 + hypertension + diabetes + kidney problems + smoking + alcohol consumption + BMI + LDL-cholesterol (all from study visit that occurred before hypertension hospitalization)

# **Table S4. Association of socioeconomic position with uncontrolled BP (SBP≥ 130 or DBP≥ 80 mmHg) at the next CARDIA visit after hospitalization using Poisson regression with robust standard errors***^†^

| Risk Ratio (95% CI) | Model 1 | Model 2 |
| --- | --- | --- |
| **Maximum educational level attained** | | |
| > High school | Reference | Reference |
| ≤ High school | 1.17 [0.70, 1.94] | 1.24 [0.72, 2.14] |
| **Family income** | | |
| ≥ $25,000 | Reference | Reference |
| < $25,000 | 1.23 [0.84, 1.79] | 1.20 [0.78, 1.85] |
| **Health insurance** | | |
| Private | Reference | Reference |
| Not having private insurance ^‡^ | 1.31 [0.89, 1.93] | 1.35 [0.90, 2.02] |
| **Neighborhood deprivation factor score – tertile 3 refers to the most socially deprived** | | |
| Tertile 1 range  [-4.07, -0.76) | Reference | Reference |
| Tertile 2 range  [-0.76, 0.23) | 0.61 [0.38, 0.98] | 0.48 [0.27, 0.84] |
| Tertile 3 range  [0.23, 2.82] | 0.75 [0.48, 1.18] | 0.79 [0.45, 1.39] |

BP: blood pressure; SBP: systolic blood pressure; DBP: diastolic blood pressure; CARDIA: Coronary Artery Risk Development in Young Adults; VA: Veterans Affairs

* This includes 46 participants who were hospitalized for hypertension between the CARDIA baseline visit and year 30 exam (2015–2016) and who had a subsequent CARDIA exam.

† Educational level, family income and insurance category were assessed at the last attended CARDIA study visit up and including year 30. Neighborhood deprivation was assessed at the last attended CARDIA study visit up and including year 20.

‡ Not having private insurance: Medicare or Medicaid or VA or no insurance

Model 1: adjust for age, sex, race, center (age at time of hypertension hospitalization, sex and center from Y0)
Model 2: Model 1 + hypertension + diabetes + kidney problems + smoking + alcohol consumption + BMI + LDL-cholesterol (all from study visit that occurred before hypertension hospitalization)

**Table S5. Incidence rate and risk ratio of cardiovascular events or kidney failure following hypertension hospitalization by socioeconomic position***^†^

|  | Developed cardiovascular event or kidney failure | Person time from hospitalization to event (in years) | Incidence rate of cardiovascular event or kidney failure/1,000 person years (95% CI) | Risk Ratio  (95% CI) ^‡^ |
| --- | --- | --- | --- | --- |
| **Maximum educational level attained** | | | | |
| > High school | 15 | 438.39 | 34.2 (16.9, 51.5) | 1 (reference) |
| ≤ High school | 8 | 165.92 | 48.2 (14.8, 81.6) | **3.93 [1.39, 11.05]** |
| **Family income** | | | | |
| ≥ $25,000 | 7 | 313.38 | 22.3 (5.8, 38.8) | 1 (reference) |
| < $25,000 | 14 | 251.23 | 55.7 (26.5, 84.9) | 1.87 [0.63, 5.55] |
| **Health insurance** | | |  |  |
| Private | 14 | 357.28 | 39.2 (18.7, 59.7) | 1 (reference) |
| Not having private insurance ^§^ | 9 | 236.69 | 38.0 (13.2, 62.9) | 1.09 [0.96, 1.26] |
| **Neighborhood deprivation factor score –tertile 3 refers to the most socially deprived** | | | | |
| Tertile 1 range  [-4.07, -0.76) | 0 | 43.99 | 0 (0, 0) | ----- |
| Tertile 2 range  [-0.76, 0.23) | 7 | 214.22 | 32.7 (8.5, 56.9) | 1 (reference) |
| Tertile 3 range  (0.23, 2.82] | 16 | 346.09 | 46.2 (23.6, 68.9) | 1.34 [0.41, 4.29] |

CARDIA: Coronary Artery Risk Development in Young Adults; CI: confidence interval
* Cardiovascular events or kidney failure ascertained and adjudicated by CARDIA

† Educational level, family income and insurance category were assessed at the last attended CARDIA study visit up and including year 30. Neighborhood deprivation was assessed at the last attended CARDIA study visit up and including year 20.

‡ Risk ratio was adjusted for age, sex, race and CARDIA site

§ Not having private insurance: Medicare or Medicaid or VA or no insurance
